# Cholangioscope combined with biliary forceps: a novel double-endoscope traction method for rectal endoscopic submucosal dissection

**DOI:** 10.1055/a-2663-8549

**Published:** 2025-08-20

**Authors:** Xia Peng, Faliang Xiang, Cheng Tang, Xuefeng Li, Rengyun Xiang

**Affiliations:** 1 74680 Department of Gastroenterology, The First Affiliated Hospital of Jishou University, Jishou, China; 2 480673 Jishou University School of Medicine, Jishou, China

Endoscopic submucosal dissection (ESD) is a technically challenging procedure 1 . Inadequate visualization of submucosal tissue planes is one of the major technical challenges. Recently, traction devices to facilitate ESD procedures have attracted an increasing interest 2 3 . Nevertheless, many traction methods are constrained by a fixed traction direction and force. Herein, we report a novel traction method—cholangioscope ( Fig. 1 **a** ) combined with biliary forceps ( Fig. 1 **b** ), which can flexibly adjust the traction direction and force.

**Fig. 1:**
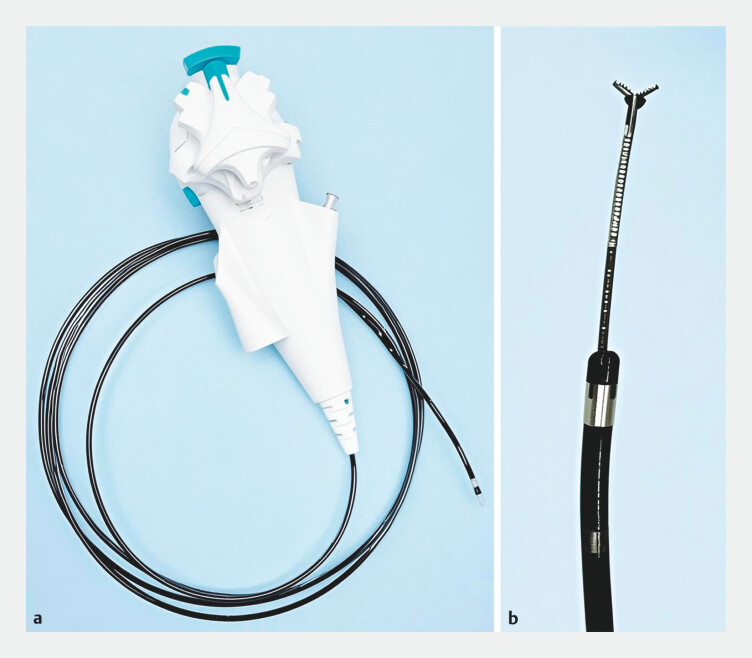
The traction device used in the ESD procedure: **a** cholangioscope and **b** biliary forceps.

A 69-year-old man presented with a 20 × 28-mm laterally spreading tumor in the rectum ( Fig. 2 **a** ), and ESD was conducted ( Video 1 ). Initially, a circumferential mucosal incision was made using a DualKnife (Anrei, Hangzhou, China) ( Fig. 2 **b** ). The cholangioscope (Leinzett, Hangzhou, China) was subsequently used to intubate the rectum and reach the lesion site, after which biliary forceps (Leinzett, Hangzhou, China) were inserted into the cholangioscope through the biopsy channel. During dissection, the proximal edge of the mucosal flap was grasped by biliary forceps, and then, the cholangioscope was adjusted to apply traction to the dissection plane ( Fig. 2 **c** ). The biliary forceps can regrasp the flap and change the traction direction and force by adjusting the cholangioscope to improve visualization of the submucosal plane and enhance exposure of the submucosal tissue ( Fig. 2 **d** ). Finally, the submucosal dissection was safely completed without any adverse events. The defect after dissection ( Fig. 2 **e** ) was closed by the use of through-the-scope clips. The total time of complete en bloc resection was 25 minutes. Histopathology revealed an adenoma with a negative margin ( Fig. 2 **f** ). In conclusion, cholangioscope combined with biliary forceps can provide adequate visualization of the submucosa during dissection and is a safe and effective traction method for the ESD procedure.

Cholangioscope combined with biliary forceps: a novel double-endoscope traction method for rectal endoscopic submucosal dissection.Video 1

**Fig. 2:**
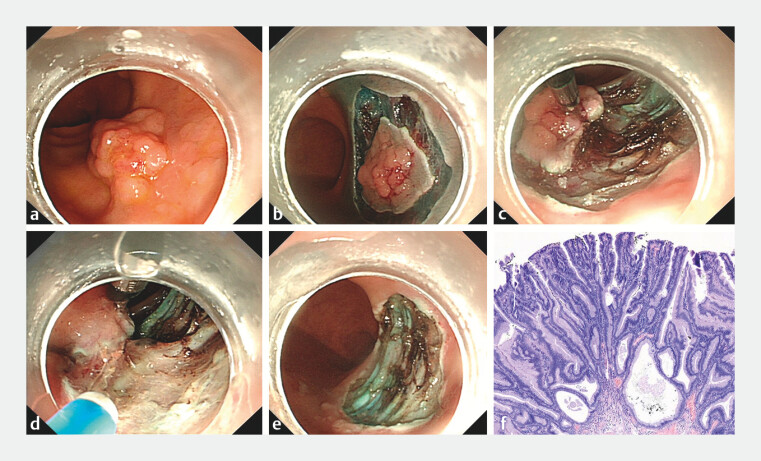
**a** A 20-mm × 28-mm laterally spreading tumor in the rectum. **b** Circumferential mucosal incision was performed. **c** The proximal edge of the mucosal flap was grasped by biliary forceps. **d** The biliary forceps regrasped the flap and changed the traction direction and force by adjusting the cholangioscope for adequate visualization of the submucosal tissue planes. **e** The defect after ESD. **f** Histopathology revealed an adenoma with a negative margin.

Endoscopy_UCTN_Code_TTT_1AQ_2AD_3AD

## Correction

In this article the authorship has been corrected. Correct is the following authorship: Xia Peng, Faliang Xiang, Cheng Tang, Xuefeng Li, Rengyun Xiang.This was corrected in the online version on August 28, 2025.

## Notes

Endoscopy E-Videos https://eref.thieme.de/e-videos
*E-Videos* is an open access online section of the journal *Endoscopy* , reporting on interesting cases and new techniques in gastroenterological endoscopy. All papers include a high-quality video and are published with a Creative Commons CC-BY license. Endoscopy E-Videos qualify for HINARI discounts and waivers and eligibility is automatically checked during the submission process. We grant 100% waivers to articles whose corresponding authors are based in Group A countries and 50% waivers to those who are based in Group B countries as classified by Research4Life (see: https://www.research4life.org/access/eligibility/ ). This section has its own submission website at https://mc.manuscriptcentral.com/e-videos .
