# Correction: Cholangioscope combined with biliary forceps: a novel double-endoscope traction method for rectal endoscopic submucosal dissection

**DOI:** 10.1055/a-2691-0593

**Published:** 2025-08-28

**Authors:** Xia Peng, Faliang Xiang, Cheng Tang, Xuefeng Li, Rengyun Xiang

**Affiliations:** 1 74680 Department of Gastroenterology, The First Affiliated Hospital of Jishou University, Jishou, China; 2 480673 Jishou University School of Medicine, Jishou, China



In the above-mentioned article the authorship has been corrected. Correct is the following authorship: Xia Peng, Faliang Xiang, Cheng Tang, Xuefeng Li, Rengyun Xiang. This was corrected in the online version on August 28, 2025.

